# Local Microstructural Environments Govern Evolution of Precipitate–Dislocation Interactions in an Al-Sc Alloy: Plasticity Regulation and Crack-Path Selection

**DOI:** 10.3390/ma19194211

**Published:** 2026-10-02

**Authors:** Bo Gao, Tuo Li, Guoqiang Ma, Zhibing Chu

**Affiliations:** 1 Analytical Test Center, Taiyuan University of Science and Technology, Taiyuan 030024, China; gaobo@tyust.edu.cn; 2 School of Materials Science and Engineering, Taiyuan University of Science and Technology, Taiyuan 030024, China; lituo@tyust.edu.cn (T.L.); guomacn@tyust.edu.cn (G.M.)

**Keywords:** Al-Sc alloy, precipitates, precipitate–dislocation interactions, in-situ TEM, crack-path deflection

## Abstract

**Highlights:**

- Nanoscale Al_3_Sc precipitates average 27.0 ± 6.3 nm with ~27.5 μm^−2^ areal density.
- Orowan bypass contributes ~67 MPa in shear and ~205 MPa in macroscopic strengthening.
- In-situ TEM reveals repeated precipitate-controlled dislocation pinning and depinning.
- Precipitate arrays direct dislocation transport and grain-boundary strain accommodation.
- Crack-tip pinning redistributes plasticity and contributes to crack-path deflection.

**Abstract:**

Nanoscale Al_3_Sc precipitates strongly influence dislocation transport and heterogeneous plastic deformation in Al-Sc alloys. Here, the environment-dependent evolution of precipitate–dislocation interactions in an Al–0.3 wt.% Sc alloy was investigated by in-situ TEM tensile testing, assisted by EBSD, TEM, EDS, and atomic-resolution HAADF-STEM. The ordered Al_3_Sc precipitates exhibit an average equivalent diameter of 27.0 ± 6.3 nm, an areal number density of ~27.5 μm^−2^, and an average center-to-center spacing of ~102 nm, corresponding to an effective obstacle spacing of ~75 nm. The modified Orowan model gives a critical shear-stress increment of ~67 MPa and a macroscopic strengthening contribution of ~205 MPa. During intragranular deformation, dislocations undergo repeated bowing, pinning, depinning, and renewed glide. Near grain boundaries, precipitate arrangements promote directional dislocation transport and heterogeneous strain accommodation. At the crack tip, selective pinning redistributes plastic flow, promotes crack-tip blunting, and activates alternative slip paths, contributing to crack-path deflection. These observations establish a continuous mechanistic link between nanoscale precipitate characteristics, dislocation transport, heterogeneous plasticity, and crack propagation in Al-Sc alloys.

## 1. Introduction

Aluminum alloys have long been extensively employed in lightweight structural applications, particularly in the aerospace and transportation industries, owing to their low density, high specific strength, good formability, and well-established manufacturing routes [1,2]. Among the various microalloying elements used to tailor the microstructure and properties of Al alloys, Sc is particularly effective: minor Sc additions can substantially enhance strength, thermal stability, and resistance to recrystallization while stabilizing the grain structure [3,4,5]. In Al-rich Al-Sc alloys, decomposition of the supersaturated solid solution during appropriate thermal treatments produces nanoscale Al_3_Sc precipitates with the ordered L1_2_ structure. These precipitates exhibit a small lattice misfit with the Al matrix, maintain a coherent interface at sufficiently small sizes, and possess high thermal stability [6,7,8,9]. Dispersed Al_3_Sc precipitates interact strongly with crystalline defects. Moving dislocations are impeded through precipitate shearing or bypass, whereas grain and subgrain boundary migration can be effectively restricted by precipitate pinning [8,9,10,11]. Consequently, the interaction of Al_3_Sc precipitates with dislocations and grain boundaries governs not only conventional precipitation strengthening but also the spatial distribution of plastic deformation at the microscale. Under sustained external loading, these interactions continuously evolve with the local deformation environment. Directly resolving the dynamic interactions among precipitates, dislocations, grain boundaries, and cracks is therefore essential for understanding plastic deformation and damage evolution in Al-Sc alloys.

Considerable progress has recently been made in understanding the influence of Sc and Sc-containing precipitates on the mechanical response of Al alloys. Nanoscale L1_2_-structured Al_3_Sc and Al_3_(Sc,Zr) precipitates provide pronounced strengthening at ambient and elevated temperatures, while their high thermal stability contributes to the retention of strength and microstructural stability; these characteristics have also promoted the development of Sc-containing high-performance Al alloys through both conventional processing and additive manufacturing routes [12,13,14,15]. At the microscopic level, dislocation–precipitate interactions depend strongly on precipitate size, coherency, and the local dislocation configuration, giving rise to pinning, shearing, Orowan bypass, and localized slip-band formation [16,17,18]. The pinning of grain and subgrain boundaries by Al_3_Sc particles further modifies dislocation accumulation, transmission, and plastic accommodation in grain-boundary-adjacent regions [10,11]. As deformation becomes increasingly localized at a crack tip, dislocation emission and glide, together with their interactions with microstructural obstacles, control the development of crack-tip plasticity, while precipitates and other secondary phases can further modify local deformation and the crack propagation path [19,20,21]. In-situ approaches have provided particularly valuable access to these transient processes. Clark et al. directly resolved dynamic dislocation–precipitate interactions and bypass processes in an Al alloy by in-situ transmission electron microscopy (in-situ TEM) [22], while Dám et al. observed active dislocation motion together with grain/subgrain-boundary evolution during in-situ deformation of an Al–Mg–Sc alloy [23]. In-situ three-dimensional X-ray tomography has further established direct correlations between microstructural heterogeneity and evolving cracks in Al alloys [24]. More recently, in-situ TEM observations demonstrated that secondary-phase-induced dislocation pinning near a crack tip can be accompanied by crack-tip blunting and crack-path deflection [25]. These studies have established key links between precipitates and individual deformation or fracture processes; however, the continuous evolution of precipitate–dislocation interactions from the grain interior through grain-boundary-adjacent regions to the crack tip, and their progressive influence on heterogeneous plasticity and crack-path selection, remains to be dynamically resolved.

Although previous studies have established important relationships between Al_3_Sc precipitates and individual deformation or fracture processes, several fundamental questions regarding their dynamic role in plastic deformation remain unresolved. In particular, it is still unclear how precipitate-mediated dislocation activities evolve when deformation progressively transfers from relatively unconstrained grain interiors to highly constrained grain-boundary regions and finally to stress-concentrated crack-tip areas.

In the present study, in-situ TEM tensile testing, combined with electron backscatter diffraction (EBSD), conventional TEM, Energy-dispersive X-ray spectroscopy (EDS), and atomic-resolution high-angle annular dark-field scanning transmission electron microscopy (HAADF-STEM), was employed to systematically investigate the dynamic precipitate–dislocation interactions in an Al–0.3 wt.% Sc alloy across distinct local microstructural environments. The initial deformation microstructure and nanoscale precipitates were first characterized from the grain scale to the atomic scale, including grain orientations, local orientation gradients, dislocation structures, and the ordered atomic configuration and elemental distribution of the precipitates. The subsequent in-situ observations were then focused on the spatial evolution of precipitate–dislocation interactions during tensile deformation. During the early stage of deformation within grain interiors, successive dislocation pinning, depinning, and continued glide at nanoscale precipitates were directly observed. In the intermediate stage, the investigation was extended to grain-boundary-adjacent regions, where precipitate-defined slip channels regulate dislocation transport toward grain boundaries and are coupled with dislocation retention and transmission at the boundaries. During the late stage, the highly stressed crack-tip region was examined to resolve the roles of precipitate–dislocation interactions in the evolution of local slip channels, redistribution of crack-tip plasticity, crack-tip blunting, and crack-path deflection. These multiscale and dynamic observations establish a continuous link between the local microstructural environment, precipitate–dislocation interactions, heterogeneous plastic deformation, and crack propagation in the Al-Sc alloy.

## 2. Materials and Methods

An Al–0.3 wt.% Sc alloy with the nominal composition was investigated in the present study. Rectangular plates with dimensions of 15mm × 10mm × 10 mm (length × width × thickness) were sectioned from the as-received billet and cold rolled along the longitudinal direction. After 15 rolling passes, the plate thickness was reduced from 10 mm to 2.5 mm. The cold-rolled plates were subsequently annealed at 300 °C for 0.5 h and used for the subsequent investigations.

**Figure 1:**
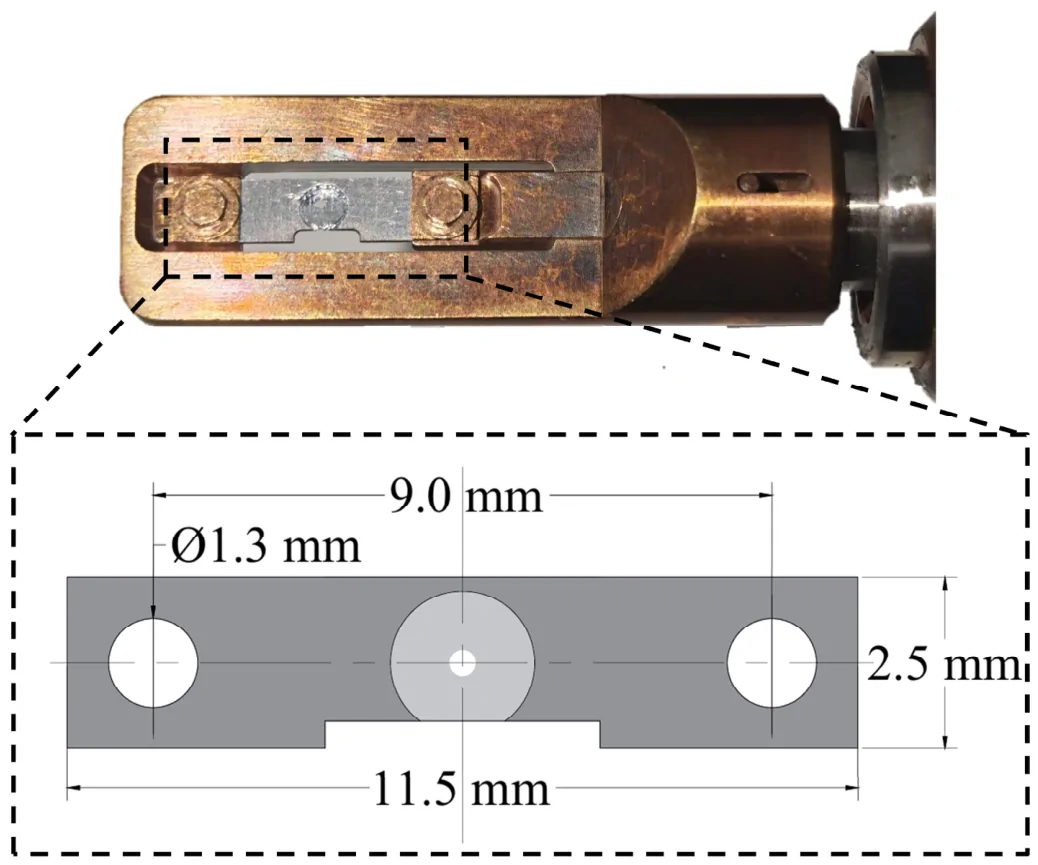
Schematic illustration of the geometry and dimensions of the tensile specimen used for the in-situ TEM tensile test, with the loading direction indicated.

The initial microstructure of the heat-treated plates was characterized on the rolling direction-normal direction (RD-ND) plane by electron backscatter diffraction (EBSD). The EBSD measurements were performed using a JSM-IT500 scanning electron microscope equipped (JEOL Ltd., Akishima, Tokyo, Japan) with an EBSD detector and operated at an accelerating voltage of 20 kV. The EBSD measurements were used to characterize the grain morphology, crystallographic orientation, and local misorientation distribution of the alloy. Specimens for in-situ TEM tensile testing were prepared by electrical discharge machining to the required geometry, followed by mechanical grinding to a thickness of approximately 100 μm. For atomic-resolution characterization, TEM specimens were mechanically ground to a thickness of approximately 30 μm and subsequently punched into 3-mm-diameter discs. The central regions of both types of TEM specimens were further thinned to electron transparency by twin-jet electropolishing. The electrolyte consisted of 10% perchloric acid and 90% ethanol, and electropolishing was performed at −30 °C under an applied voltage of 20 V. In-situ TEM tensile experiments were conducted using a JEM-F200 transmission electron microscope (JEOL Ltd., Akishima, Tokyo, Japan) operated at an accelerating voltage of 200 kV. Tensile loading was applied using a Gatan 671 single-tilt liquid-nitrogen cooling tensile holder (Gatan, Inc., Warrendale, USA) at a constant displacement rate of 2.0 μm s^−1^. All in-situ tensile experiments were performed at room temperature without liquid-nitrogen cooling. The time stamps shown in the in-situ TEM image sequences denote the elapsed recording time during continuous displacement-controlled tensile loading and do not represent holding time under a constant load. The specimen was subjected to uniaxial tensile loading throughout the experiment. The geometry of the tensile specimen and its configuration in the tensile holder are schematically illustrated in Figure 1. The dynamic evolution of dislocation motion, precipitate–dislocation interactions, and microstructural changes in the crack-tip region was continuously recorded during in-situ loading. Atomic-scale structural characterization of the precipitates was performed by HAADF-STEM using an aberration-corrected JEM-ARM300F2 electron microscope (JEOL Ltd., Akishima, Tokyo, Japan), enabling direct examination of the atomic arrangement and local structural characteristics of the nanoscale precipitates.

## 3. Results

**Figure 2:**
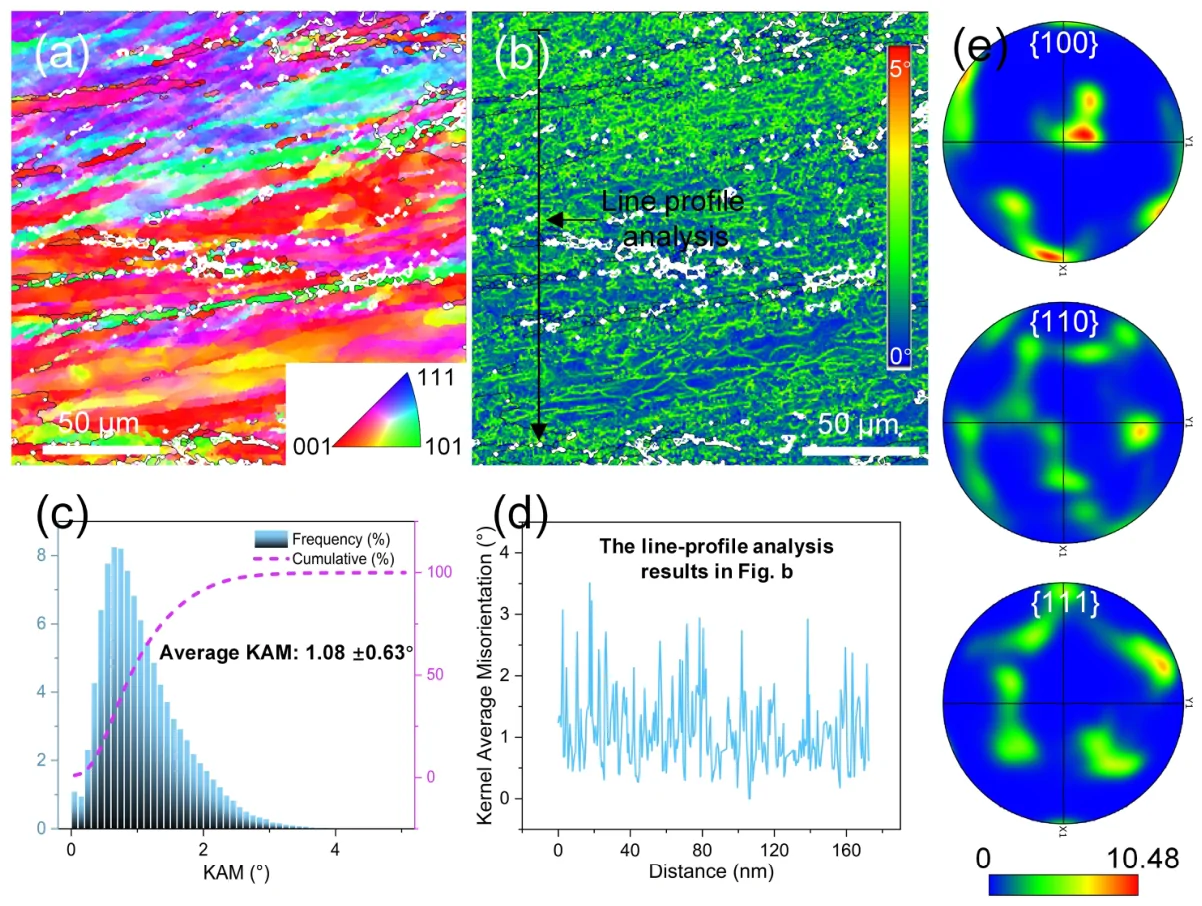
EBSD characterization of the initial microstructure of the Al-0.3 wt.% Sc alloy: (**a**) inverse pole figure (IPF) orientation map acquired on the RD–ND plane; (**b**) corresponding kernel average misorientation (KAM) map, with the line used for profile analysis indicated; (**c**) frequency and cumulative distributions of KAM values; (**d**) KAM line-profile analysis along the marked path in (**b**), showing the spatial variation in local misorientation; (**e**) corresponding {100}, {110}, and {111} pole figures showing the crystallographic texture of the initial microstructure.

Figure 2 presents the EBSD characterization of the initial microstructure of the Al–0.3 wt.% Sc alloy. As shown in Figure 2a, the grains are distinctly elongated along the rolling direction (RD), indicating that the deformation morphology introduced by cold rolling is largely retained after the subsequent short-term heat treatment at 300 °C. In addition, pronounced crystallographic orientation differences are observed both between neighboring grains and within individual grains. The corresponding KAM map in Figure 2b shows that relatively high local misorientation values are mainly concentrated near grain boundaries and in some intragranular regions, indicating that a certain degree of local lattice distortion is retained after heat treatment. The quantitative KAM distribution in Figure 2c further demonstrates that most KAM values are concentrated below approximately 2°, with only a small fraction extending to higher misorientation ranges, with an average KAM value of 1.08 ± 0.63° (mean ± standard deviation). The line-profile analysis in Figure 2d further confirms the spatial fluctuation of local misorientation, with several localized peaks reaching approximately 3.5°. In addition, the {100}, {110}, and {111} pole figures in Figure 2e exhibit pronounced orientation intensity concentrations, indicating that significant rolling-related texture is still retained after the short-term annealing treatment.

**Figure 3:**
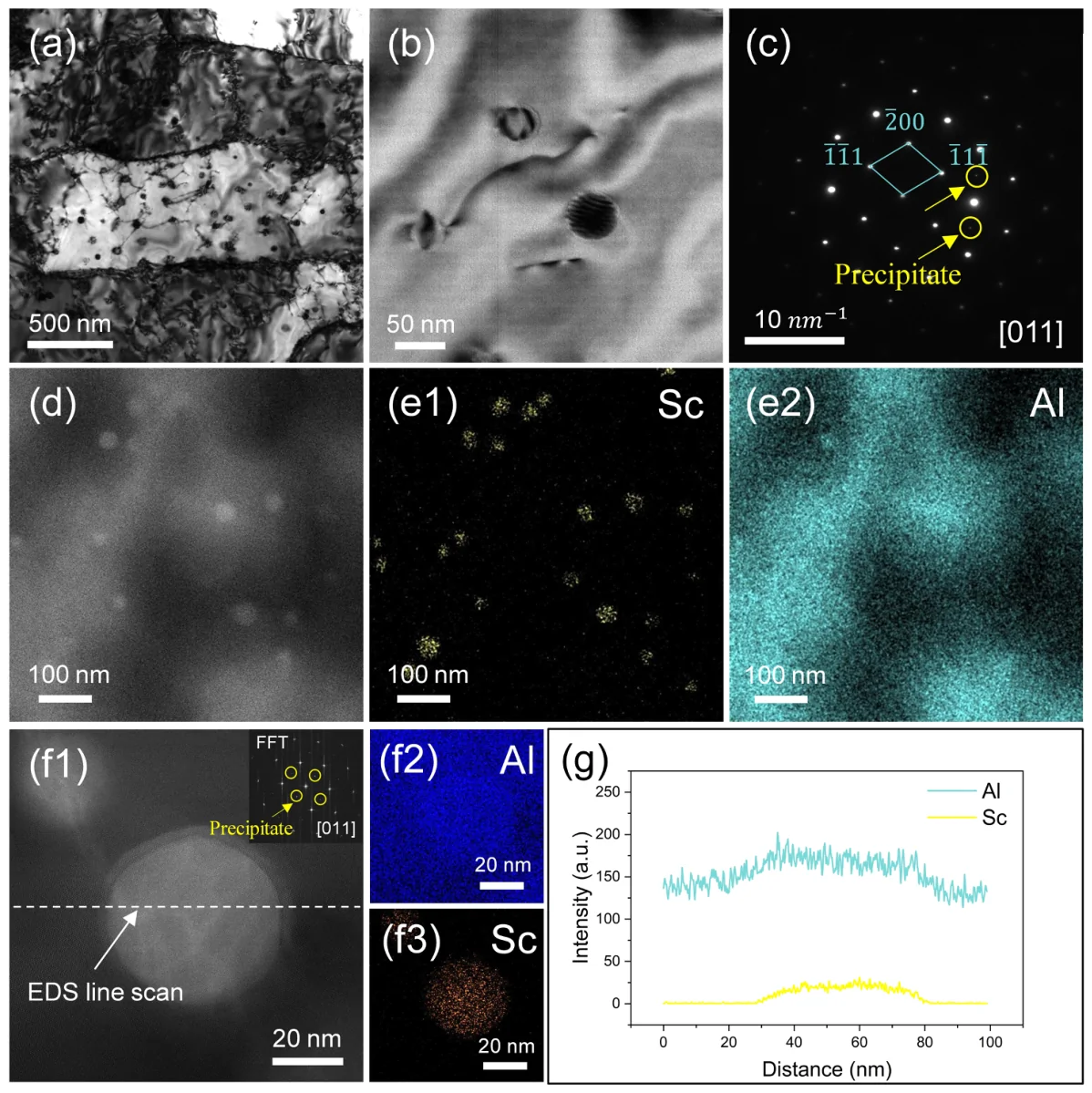
TEM characterization of dislocations and nanoscale precipitates in the initial microstructure of the Al–0.3 wt.% Sc alloy: (**a**) low-magnification bright-field TEM image; (**b**) enlarged TEM image showing a representative precipitate–dislocation interaction; (**c**) diffraction pattern acquired along the [011] zone axis, with the precipitate-related superlattice reflections highlighted; (**d**) STEM image of the precipitate-containing region and corresponding EDS elemental maps of (**e1**) Sc and (**e2**) Al; (**f1**) aberration-corrected atomic-resolution image of an individual precipitate with the corresponding fast Fourier transform (FFT) pattern, and elemental maps of (**f2**) Al and (**f3**) Sc; (**g**) EDS line-scan profiles of Al and Sc across the individual precipitate.

Figure 3 presents the TEM characterization of the initial dislocation structure and nanoscale precipitates in the Al-0.3 wt.% Sc alloy. As shown in Figure 3a, a high number density of nanoscale precipitates is distributed within the elongated grains and near grain-boundary regions, while a considerable density of dislocations is retained in the Al matrix. Local bending, accumulation, and tangling of dislocations are frequently observed around the particles, indicating that the precipitates already interact strongly with the retained dislocation network before tensile loading. The enlarged image in Figure 3b further reveals pronounced dislocation curvature in the vicinity of an individual precipitate, providing direct evidence of local precipitate-induced dislocation pinning.

The diffraction pattern obtained along the [011] zone axis in Figure 3c contains, in addition to the fundamental reflections of the Al matrix, weak additional reflections associated with the precipitates. These superlattice reflections are characteristic of an ordered structure. The STEM image and corresponding elemental maps in Figure 3(d,e1,e2) show that Sc is preferentially enriched at the nanoscale particles, whereas Al is continuously distributed throughout the matrix and precipitate-containing regions, confirming the formation of Sc-rich Al-Sc precipitates. To further determine the structure and composition of an individual precipitate, representative particles were characterized at high spatial resolution, as shown in Figure 3(f1–f3). The aberration-corrected atomic-resolution image and corresponding FFT pattern in Figure 3(f1) reveal superlattice reflections along the [011] zone axis. The corresponding EDS elemental maps show pronounced Sc enrichment within the particle, whereas Al is broadly distributed throughout both the matrix and the precipitate-containing region. The EDS line scan in Figure 3g further confirms that the Sc signal increases markedly within the precipitate and rapidly decreases to nearly the matrix background level after the probe leaves the particle. Based on the combined evidence of the superlattice reflections and localized Sc enrichment, the nanoscale precipitate can be identified as an Al_3_Sc phase.

**Figure 4:**
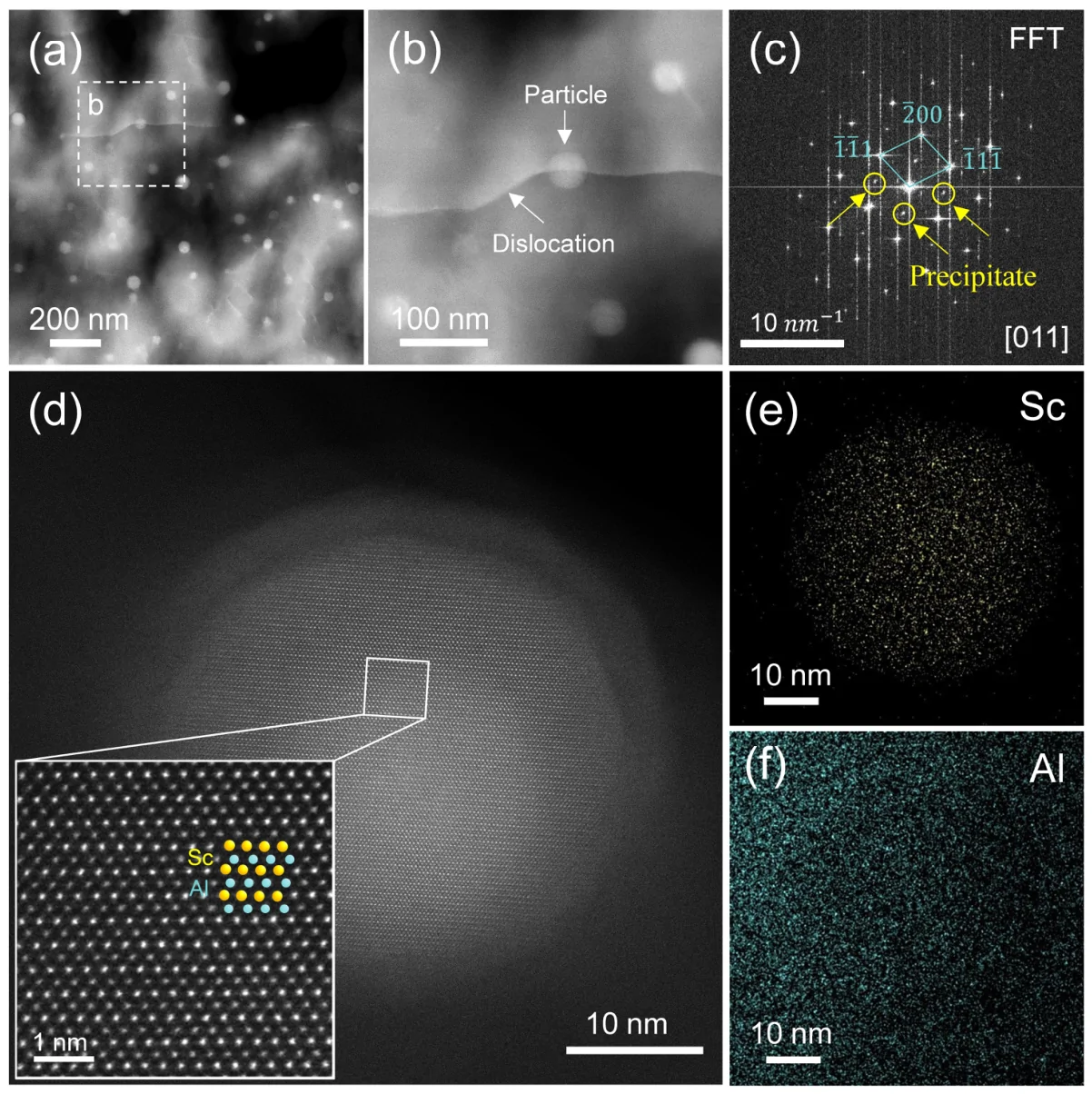
HAADF-STEM and atomic-scale characterization of nanoscale precipitates in the Al-Sc alloy: (**a**) low-magnification HAADF-STEM image; (**b**) enlarged view of the dashed region in (**a**); (**c**) FFT pattern corresponding to the atomic-resolution image in (**d**); (**d**) atomic-resolution HAADF-STEM image of an individual precipitate, revealing an ordered atomic arrangement and periodic distribution of Sc atomic columns; (**e**,**f**) corresponding EDS elemental maps of Sc and Al, respectively.

To further resolve the structural characteristics of the nanoscale precipitates in the Al-Sc alloy, HAADF-STEM was performed, as shown in Figure 4. Figure 4a presents a low-magnification HAADF-STEM image, revealing a high number density of nanoscale precipitates dispersed throughout the Al matrix. Figure 4b shows an enlarged view of the dashed region in Figure 4a, where a dislocation directly interacts with a precipitate and undergoes pronounced local bending in the vicinity of the particle.

Figure 4c presents the FFT pattern corresponding to the atomic-resolution image in Figure 4d. In addition to the fundamental reflections of the Al matrix, additional reflections associated with the precipitate are clearly observed, consistent with the diffraction characteristics described above. Figure 4d shows an atomic-resolution HAADF-STEM image of an individual precipitate. A highly ordered periodic atomic arrangement is resolved within the particle, with Sc atomic columns occupying ordered sublattice positions within the Al framework, giving rise to a distinct chemically ordered structure. The corresponding EDS elemental maps in Figure 4e,f show pronounced Sc enrichment within the particle, whereas Al is distributed throughout both the matrix and the precipitate region.

**Figure 5:**
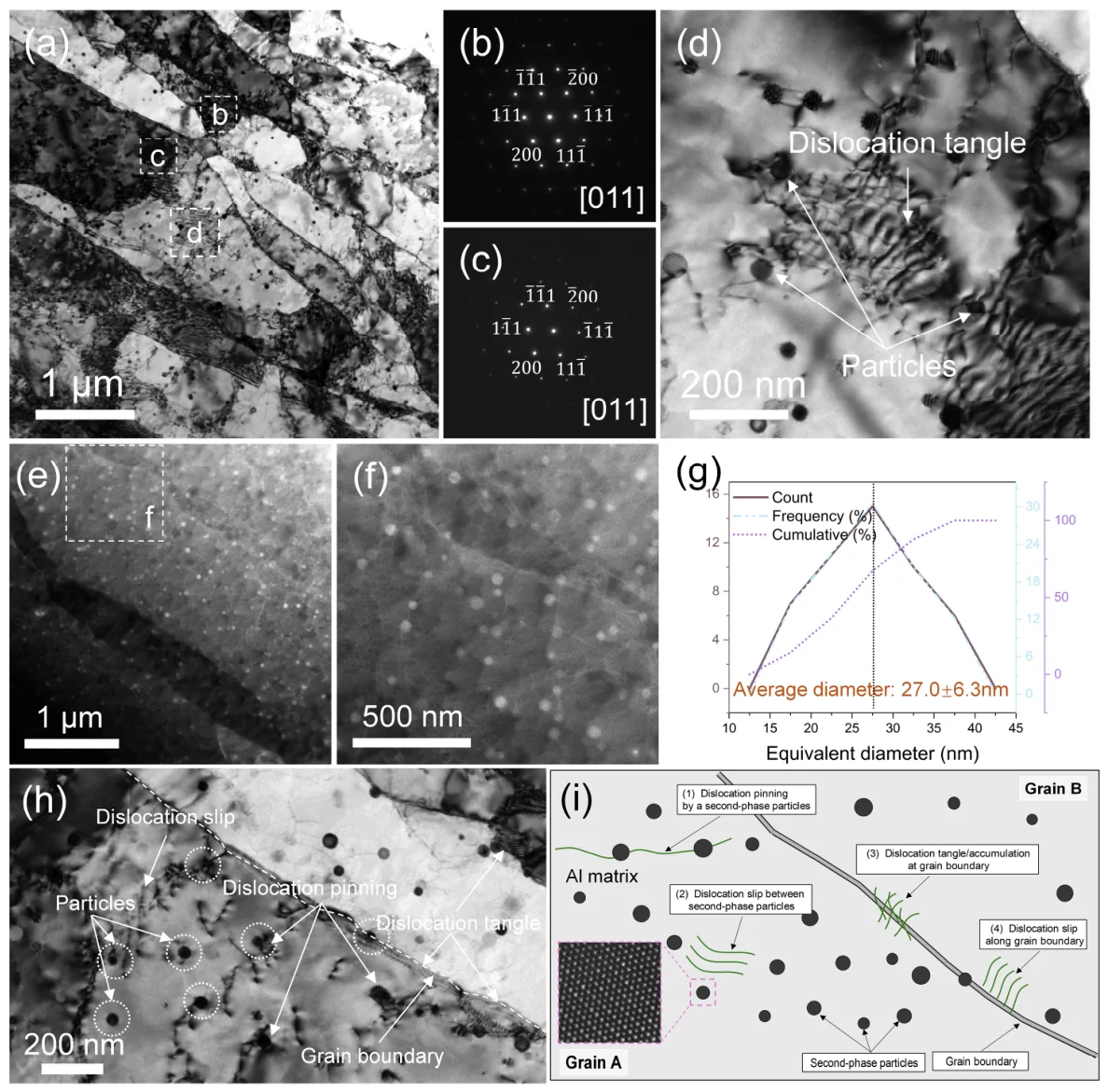
TEM/STEM characterization of the initial microstructure and precipitate–dislocation interactions in the Al–0.3 wt.% Sc alloy: (**a**) low-magnification bright-field TEM image showing elongated grains; (**b**,**c**) FFT/diffraction patterns obtained from two adjacent grains; (**d**) enlarged TEM image showing local dislocation bowing, pinning, and tangling around precipitates; (**e**) STEM image of the precipitate-containing region; (**f**) enlarged STEM image used for quantitative precipitate analysis; (**g**) statistical distribution of precipitate equivalent diameter, yielding an average size of 27.0 ± 6.3 nm; (**h**) representative precipitate–dislocation interactions near a grain boundary; (**i**) schematic illustration summarizing dislocation pinning, glide between precipitates, tangling/accumulation at the grain boundary, and slip along the grain boundary.

Figure 5 further characterizes the crystallographic orientation, precipitate size and spatial distribution, and precipitate–dislocation interactions in the initial microstructure of the Al–0.3 wt.% Sc alloy. As shown in Figure 5a, the microstructure mainly consists of grains elongated along the rolling direction, with a high density of retained dislocations and numerous nanoscale precipitates dispersed within the grains. Figure 5b,c show the FFT/diffraction patterns obtained from two adjacent grains. The clear difference in the spatial orientation of the diffraction patterns indicates a crystallographic misorientation between the neighboring grains.

Figure 5d provides an enlarged view of a representative precipitate–dislocation interaction region, where pronounced dislocation bowing, pinning, and local tangling are observed around nanoscale precipitates, demonstrating their effective resistance to dislocation motion. Figure 5e,f show the STEM morphology and an enlarged precipitate-containing region, revealing a dispersed distribution of nearly spherical nanoscale precipitates in the Al matrix. For quantitative size analysis, individual precipitates were identified from the STEM image, and their equivalent circular diameters were calculated from the two-dimensional projected areas. As summarized in Figure 5g, the precipitates exhibit an average equivalent diameter of 27.0 ± 6.3 nm, with most particles distributed within approximately 20–35 nm. Based on 49 identified precipitates within an effective analyzed area of 1.779 μm^2^, the corresponding areal number density is approximately 27.5 μm^−2^, with a projected area fraction of approximately 1.66%. Nearest-neighbor analysis gives an average center-to-center precipitate spacing of approximately 102 nm. After accounting for the average precipitate diameter, the effective obstacle spacing relevant to dislocation motion is estimated to be approximately 75 nm.

Figure 5h further illustrates several representative interaction modes between precipitates, dislocations, and the grain boundary, including dislocation pinning by individual precipitates, dislocation glide between neighboring particles, dislocation tangling and accumulation near the grain boundary, and dislocation glide along the boundary. These characteristic processes are schematically summarized in Figure 5i. Overall, the Al_3_Sc precipitates regulate dislocation transport through both their nanoscale obstacle effect and spatial distribution, while their coupling with the grain boundary further contributes to heterogeneous dislocation transport and local plastic deformation.

**Figure 6:**
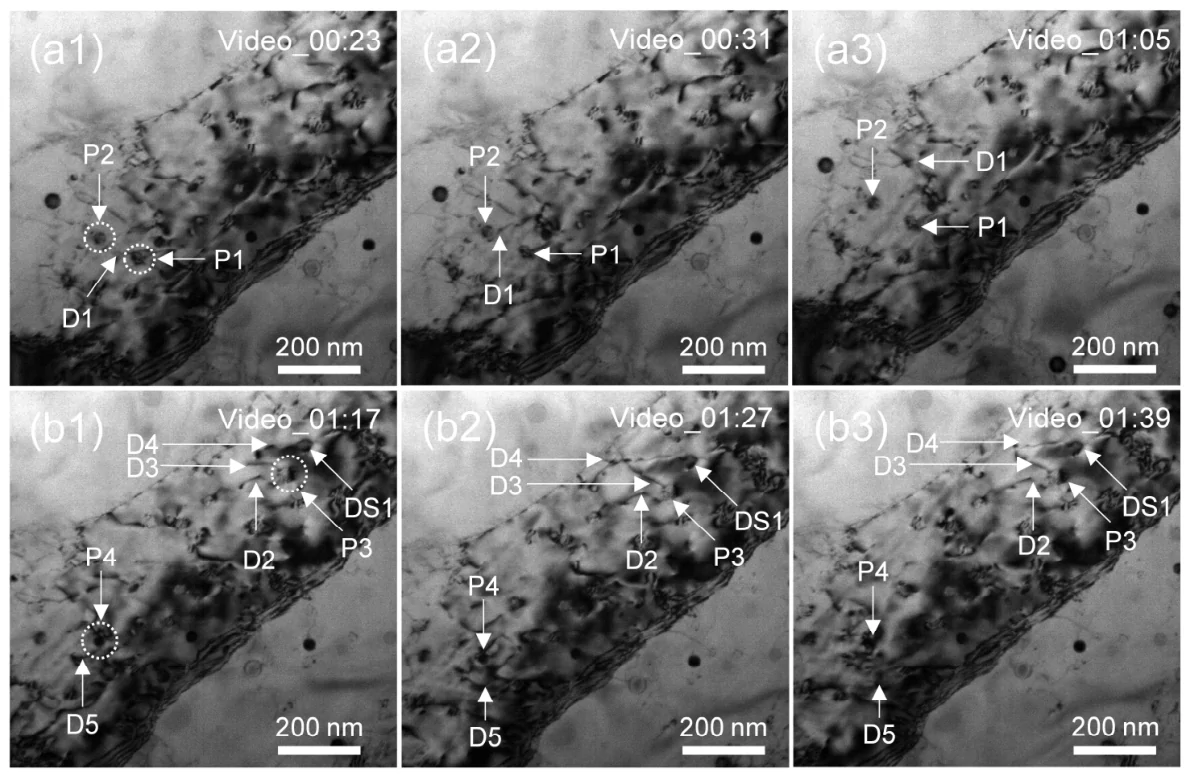
In-situ TEM observations of dynamic precipitate–dislocation interactions within the grain interior during the early stage of deformation in the Al–0.3 wt.% Sc alloy. (**a1**–**a3**) Sequential interaction of dislocation D1 with precipitates P1 and P2; (**b1**–**b3**) Evolution of multiple dislocations around precipitates P3 and P4.

To further observe the dynamic interactions between precipitates and dislocations within grain interiors during the early stage of plastic deformation in the Al-Sc alloy, in-situ TEM tensile testing was performed, as shown in Figure 6. Figure 6(a1–a3) show the successive interaction of dislocation D1 with precipitates P1 and P2. D1 is initially pinned in the vicinity of P1 and subsequently depins under continued loading, after which it glides toward P2 and is temporarily impeded again. With further deformation, D1 escapes from the vicinity of P2 and resumes motion along the original slip direction. This sequence directly demonstrates the repeated pinning–depinning and renewed glide of a moving dislocation as it encounters successive nanoscale precipitates.

Figure 6(b1–b3) further reveal the collective evolution of multiple dislocations around precipitates P3 and P4. D2 and D3 are initially located near P3, while dislocation source DS1 becomes activated and emits a new dislocation D4. Meanwhile, D5 remains pinned near P4. With continued deformation, D2 exhibits relatively limited displacement, whereas D3 interacts with the newly emitted D4 and undergoes local rearrangement before further motion. At the same time, D5 gradually depins from P4 and migrates toward the grain-boundary direction. These observations demonstrate that nanoscale precipitates locally regulate dislocation mobility through repeated pinning and depinning, while also contributing to heterogeneous dislocation accumulation and source activation within the grain interior.

**Figure 7:**
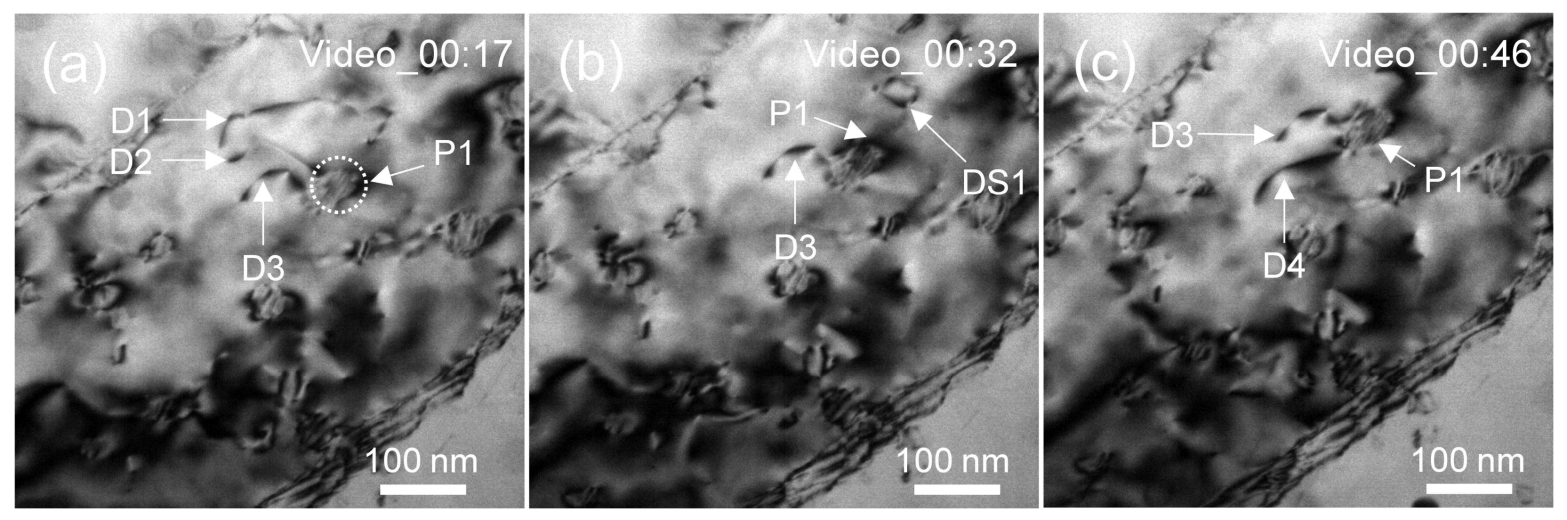
In-situ TEM observations of dynamic precipitate–dislocation interactions within the grain interior during the early stage of plastic deformation in the Al-Sc alloy: (**a**) distribution of dislocations D1, D2 and D3 around precipitate P1 at 00:17; (**b**) at 00:32, D1 and D2 have moved away from their initial positions, whereas D3 remains localized near the particle, accompanied by the activation of DS1; (**c**) at 00:46, newly generated dislocation D4 undergoes glide, while D3 remains confined in the vicinity of precipitate P1 under persistent pinning.

Figure 7 further illustrates the dynamic interactions between precipitates and dislocations within the grain interior during the early stage of plastic deformation. In Figure 7a, several dislocations are distributed in the vicinity of precipitate P1. Dislocations D1 and D2 are located above the particle, whereas D3 remains close to P1 and is impeded by the precipitate. With continued in-situ tensile loading, D1 and D2 progressively glide forward along their slip directions and subsequently leave the present field of view. In contrast, D3 remains localized in the vicinity of the precipitate throughout the sequence, with no apparent change in its position, exhibiting persistent local pinning.

Notably, at 00:32, as shown in Figure 7b, D1 and D2 have disappeared from their initial positions, whereas D3 remains adjacent to the particle. Meanwhile, dislocation source DS1 becomes active in the same region, accompanied by continued local dislocation activity. Upon further loading to 00:46, as shown in Figure 7c, the newly generated dislocation D4 glides forward, while D3 remains confined in the vicinity of precipitate P1 and its motion continues to be restricted. Distinct responses of individual dislocations to precipitate obstacles are therefore observed within the same region: some dislocations continue to glide under sustained loading and leave the observation area, whereas others remain persistently pinned in the vicinity of the particles. The nanoscale precipitates within the grain interior continuously impede and pin moving dislocations, thereby restricting dislocation glide and providing a pronounced precipitation-strengthening effect during the early stage of plastic deformation.

**Figure 8:**
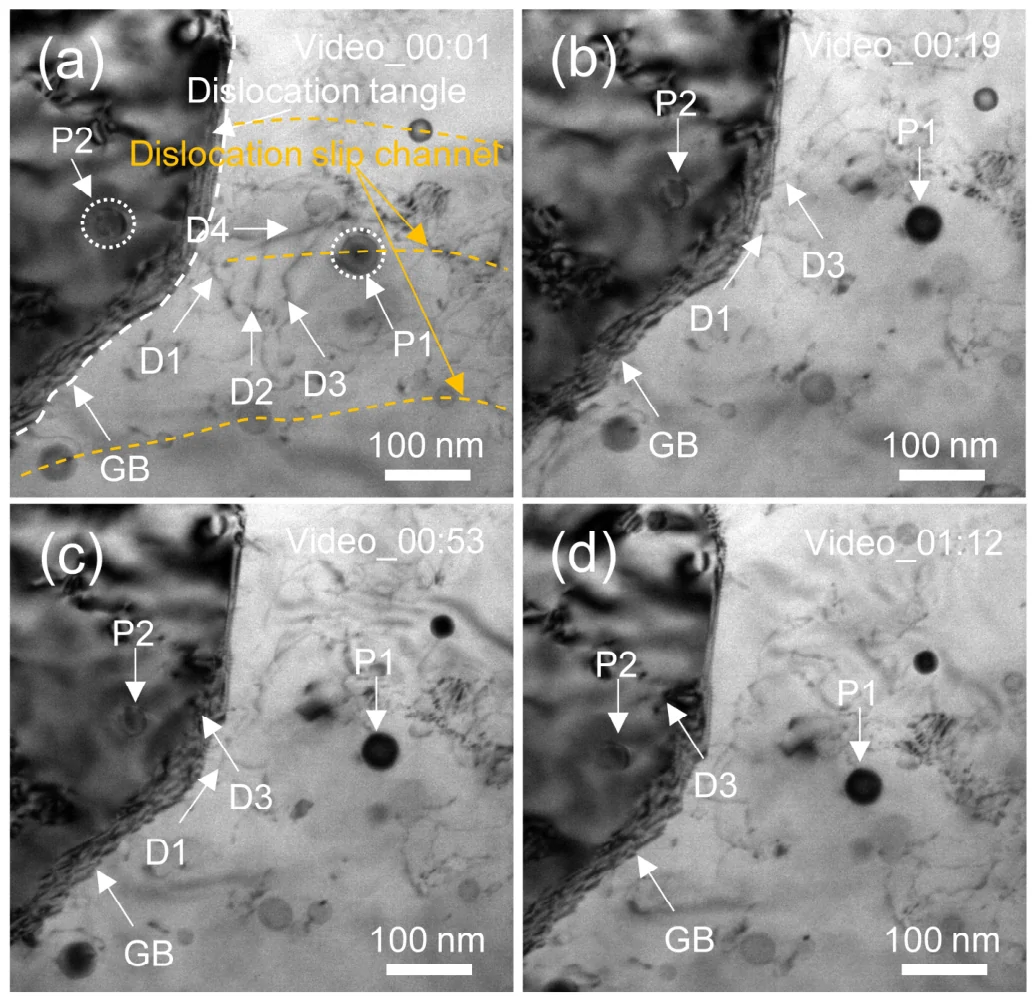
In-situ TEM observations of dislocation evolution in a grain-boundary-adjacent region during the intermediate stage of plastic deformation in the Al–0.3 wt.% Sc alloy: (**a**) initial distribution of precipitates P1 and P2 and dislocations D1–D4 near the grain boundary, showing precipitate-defined slip channels and pronounced dislocation tangling at the boundary; (**b**,**c**) progressive migration of D1 and D3 toward the grain boundary and their interaction with the pre-existing boundary dislocation structure; (**d**) subsequent transmission of D3 across the grain boundary during continued loading.

Figure 8 presents the dynamic evolution of dislocations in a grain-boundary-adjacent region during the intermediate stage of tensile deformation. As shown in Figure 8a, precipitates P1 and P2 are distributed on opposite sides of the grain boundary, while dislocations D1–D4 are located within the neighboring grain interior. The spatial arrangement of the precipitates delineates preferential slip channels, within which the motion of dislocations is largely confined. Meanwhile, pronounced dislocation tangling is already present along the grain boundary, indicating that the boundary acts as an important obstacle to dislocation transport.

With continued loading, D1 progressively migrates toward the grain boundary along the precipitate-defined slip path and interacts with the pre-existing boundary dislocation structure, resulting in local dislocation accumulation and entanglement. D2 and D4 also move toward the boundary and are no longer distinguishable in the subsequent images, whereas D3 continues to approach the boundary along the same localized slip region. As deformation proceeds, D3 becomes temporarily arrested near the grain boundary and subsequently transmits into the adjacent region, while the locally accumulated dislocation structure remains concentrated near the boundary. These observations indicate that the spatial distribution of nanoscale precipitates constrains the available dislocation glide paths and promotes directional transport toward selected grain-boundary regions. The grain boundary then further regulates the arriving dislocations through temporary arrest, entanglement, and transmission, resulting in a pronounced heterogeneous distribution of local plastic deformation.

**Figure 9:**
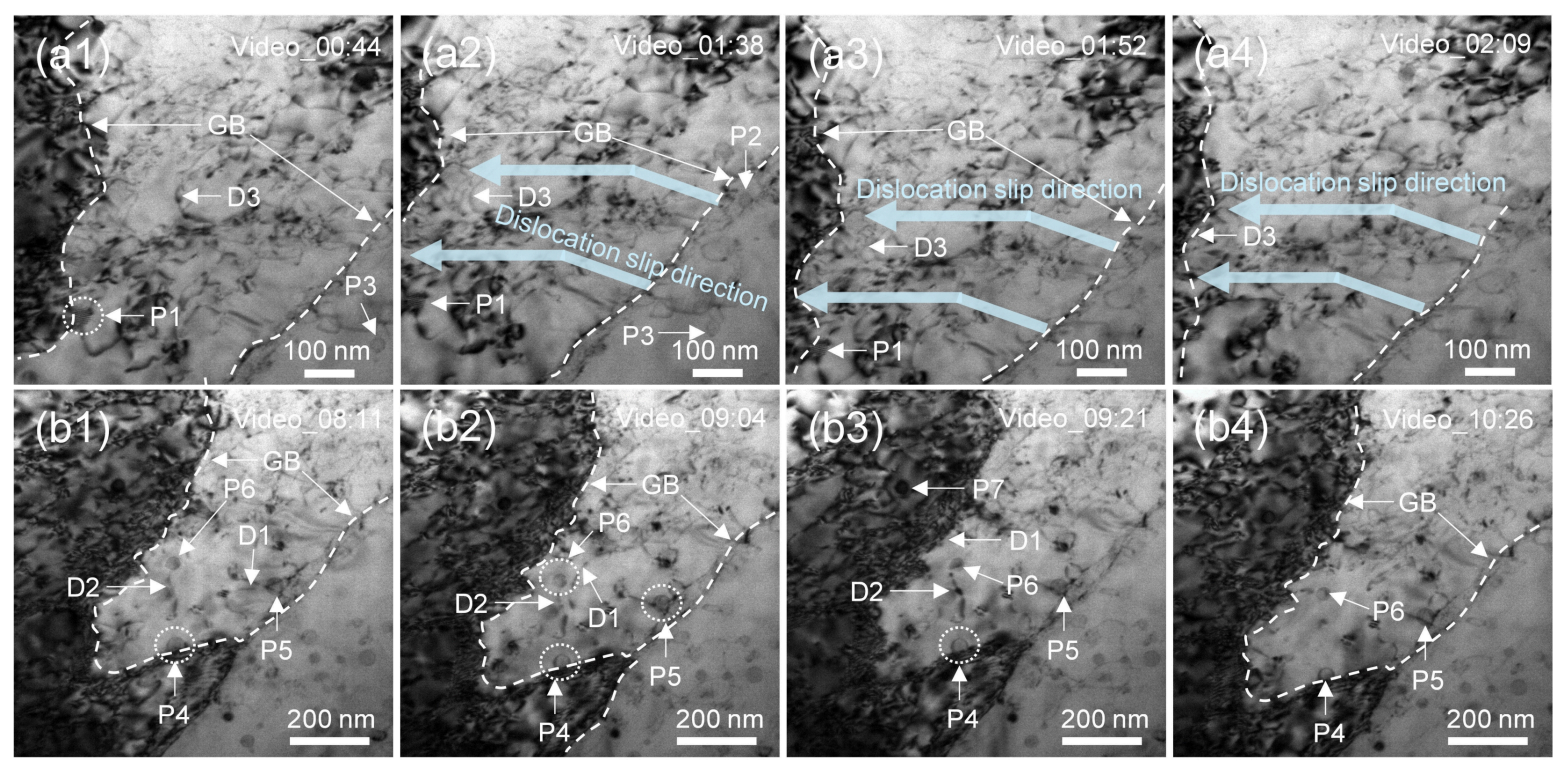
In-situ TEM observations of dynamic precipitate–dislocation interactions in grain-boundary-adjacent regions during the intermediate stage of plastic deformation in the Al-Sc alloy: (**a1**) initial distribution of precipitates P1 and P3 near the grain boundaries and dislocation D3 within the grain interior; (**a2**–**a4**) development of two preferential dislocation slip paths and progressive glide of D3 toward the left grain boundary; (**b1**) initial distribution of precipitates P4–P6 near the grain boundary, precipitate P7 within the grain interior, and dislocations D1 and D2; (**b2**–**b4**) continuous glide of D1 toward the left grain boundary and pinning, depinning, and subsequent motion of D2 from P6 toward the right grain boundary.

Figure 9 further presents the dynamic precipitate–dislocation interactions in grain-boundary-adjacent regions during the intermediate stage of plastic deformation. In Figure 9(a1), precipitate P1 is located at the left grain boundary, whereas P3 is distributed near the boundary on the opposite side. A substantial population of dislocations is present within the grain interior, among which dislocation D3 is gliding from the grain interior toward the left grain boundary. With continued in-situ tensile loading, as shown in Figure 9(a2–a4), two sets of preferential dislocation slip paths with different orientations develop within the grain, along which dislocations are transported toward the surrounding grain boundaries. D3 continuously migrates along one of these slip paths toward the left grain boundary, with its distance from the boundary progressively decreasing, and is no longer discernible in the subsequent TEM images after reaching the grain-boundary region. Meanwhile, other dislocations continue to move along their respective slip directions, producing pronounced directional and spatial heterogeneity in the local dislocation activity.

Figure 9(b1–b4) show another dynamic interaction sequence within a grain-boundary-adjacent region. In the initial state, precipitates P4 and P5 are located near the grain boundary, P6 lies within the grain at a short distance from the boundary, whereas P7 is positioned further inside the grain. Dislocations D1 and D2 are distributed within this region and exhibit distinct dynamic responses during loading. Dislocation D1 continuously glides toward the left grain boundary, progressively approaches the boundary, and is no longer discernible after reaching the grain-boundary region. In parallel, D2 is initially pinned by precipitate P6, resulting in a temporary interruption of its glide. With further tensile loading, D2 depins from P6 and resumes motion, subsequently propagating toward the right grain boundary before becoming no longer discernible after reaching the boundary. Compared with the precipitate-dominated impediment of dislocation glide observed within grain interiors during the early deformation stage, precipitate-mediated local regulation becomes increasingly coupled with grain-boundary constraints in the intermediate stage, giving rise to a more spatially partitioned and heterogeneous distribution of plastic deformation.

**Figure 10:**
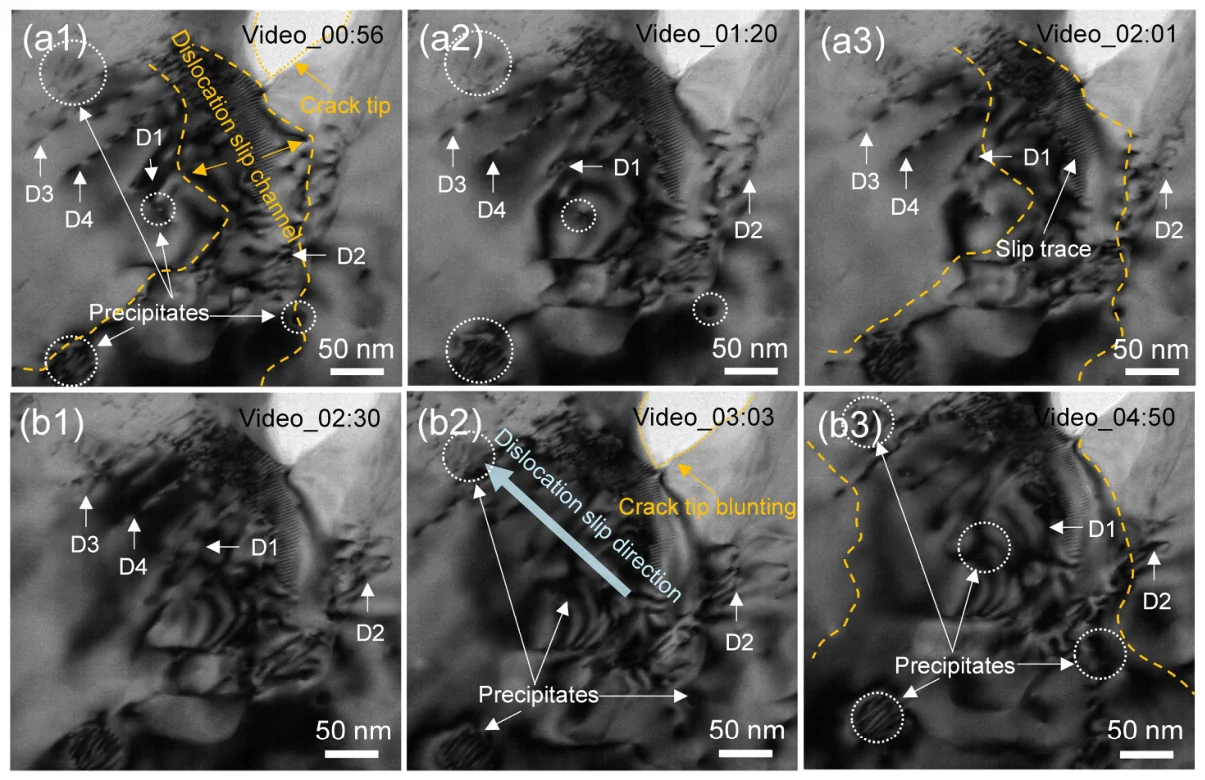
In-situ TEM observations of dynamic precipitate–dislocation interactions and crack-tip evolution during the late stage of plastic deformation in the Al-Sc alloy: (**a1**) initial distribution of precipitates, dislocations D1–D4, and the precipitate-defined dislocation slip channel ahead of the crack tip; (**a2**,**a3**) depinning and continued glide of D1 together with forward motion of D2, while D3 and D4 remain nearly stationary, accompanied by the development of a distinct slip trace ahead of the crack tip; (**b1**–**b3**) continued glide of D1 and D2 under sustained loading, persistent confinement of D3 and D4, progressive widening of the dislocation slip channel, and concurrent crack-tip blunting.

Figure 10 presents the dynamic interactions among precipitates, dislocation motion, and crack-tip morphology during the late stage of plastic deformation. As shown in Figure 10(a1), multiple nanoscale precipitates are distributed ahead of the crack tip, and their spatial arrangement delineates the boundaries of a local dislocation slip channel. A high density of dislocations is present in the crack-tip region, with D1–D4 distributed within and around the slip channel. Dislocation D1 is located in the vicinity of a precipitate and is locally pinned; D2 lies on the opposite side of the crack tip, whereas D3 and D4 are positioned outside the main slip channel. The local impediment imposed by the precipitates produces a pronounced spatial heterogeneity in dislocation activity ahead of the crack tip. With continued in-situ tensile loading, as shown in Figure 10(a2,a3), D1 progressively depins from the precipitate and glides forward along its slip direction, while D2 also continues to propagate. In contrast, D3 and D4 remain nearly stationary, with no pronounced long-range motion observed during this interval. The distinct mobility of individual dislocations progressively localizes plastic deformation within the precipitate-defined slip channel. By 02:01, a clear slip trace develops ahead of the crack tip, associated with the accumulation of local plastic deformation generated by continued dislocation glide.

Figure 10(b1–b3) further capture the subsequent evolution of dislocation activity and crack-tip morphology. As loading proceeds from 02:30 to 04:50, D1 and D2 continue to glide along their respective slip directions, whereas D3 and D4 remain largely confined to their local positions. Meanwhile, the initially localized dislocation slip region progressively expands, accompanied by a pronounced widening of the slip channel delineated by the surrounding precipitates. At 03:03, the crack tip develops an increased local curvature and becomes visibly blunted, followed by further spreading of plastic deformation over a broader region around the crack tip. In contrast to the predominantly local impediment of dislocation glide observed within grain interiors, precipitate–dislocation interactions near the crack tip increasingly regulate the spatial distribution of crack-tip plasticity and modify the local stress and deformation fields.

**Figure 11:**
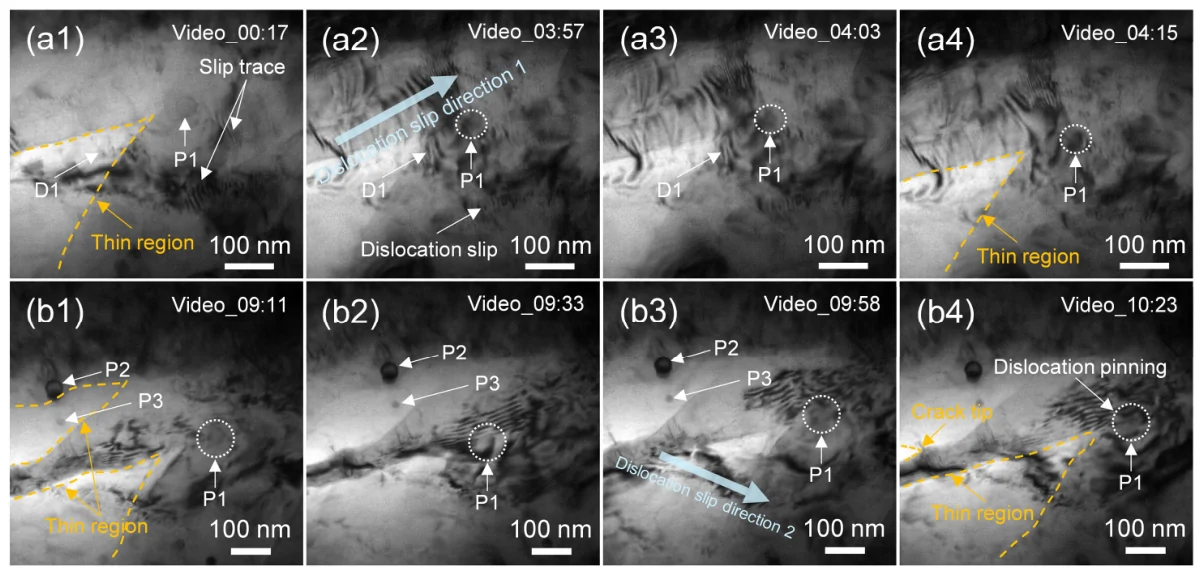
In-situ TEM observations of precipitate–dislocation interactions and crack-path deflection ahead of the crack tip during the late stage of plastic deformation in the Al-Sc alloy: (**a1**) initial thin region ahead of the crack tip, precipitate P1, dislocation D1, and associated slip traces; (**a2**–**a4**) continuous dislocation motion along slip direction 1 toward P1, followed by local impediment near the precipitate and arrest of the leading edge of the original thin region; (**b1**–**b3**) formation of a new thin region above the original one, with precipitates P2 and P3 located nearby, repeated dislocation pinning–depinning around P1, and development of a second dislocation slip direction between the two thin regions; (**b4**) preferential crack propagation into the newly formed upper thin region, while extension of the lower thin region remains restricted near P1, resulting in crack-path deflection.

Figure 11 further presents the dynamic interactions among precipitates, dislocation motion, and crack propagation within the thin region ahead of the crack tip during the late stage of plastic deformation. As shown in Figure 11(a1), a localized thin region develops ahead of the crack tip, with precipitate P1 located at its leading edge. Numerous slip traces are visible in the vicinity of P1, while dislocation D1 continuously migrates forward within the thin region, revealing pronounced localization of plastic deformation ahead of the crack tip. With continued in-situ tensile loading, as shown in Figure 11(a2–a4), a distinct dislocation slip direction, denoted as slip direction 1, develops ahead of the thin region. Dislocations continuously propagate along this direction toward P1. Upon reaching the vicinity of P1, their motion is strongly impeded and local dislocation retention develops around the particle. Dislocation D1 similarly approaches P1 along this slip direction and eventually remains confined near the precipitate. Despite sustained dislocation activity ahead of the crack tip, the leading edge of the original thin region shows little further extension beyond P1, while plastic activity becomes increasingly concentrated in its vicinity.

Upon further loading, as shown in Figure 11(b1–b4), a new thin region progressively develops above the original one, with precipitates P2 and P3 located near its local boundaries. Meanwhile, repeated dislocation pinning–depinning events continue around P1, and the crack-tip dislocation activity is no longer restricted to the initial slip direction. A second dislocation slip direction subsequently develops between the two thin regions, along which dislocations undergo continued motion. Consequently, the localized plastic deformation ahead of the crack tip progressively spreads from the original lower thin region toward the newly formed upper region. During subsequent loading, the upper thin region continues to develop, whereas the leading edge of the original lower thin region remains locally arrested near P1. The crack tip eventually propagates preferentially into the newly formed upper thin region, while forward extension of the lower region remains restricted by the persistent precipitate–dislocation interactions around P1, resulting in a pronounced deflection of the crack propagation path. It should be noted that these observations were obtained from an electron-transparent TEM foil, where the reduced through-thickness constraint and free-surface effects differ from bulk crack-tip conditions. Therefore, the observed crack-path deflection should be interpreted as a local thin-foil mechanism associated with precipitate-mediated plasticity redistribution, rather than as a direct quantitative prediction of bulk fracture behavior.

## 4. Discussion

### 4.1. Precipitate Pinning Governs Intragranular Dislocation Transport

During intragranular deformation, individual dislocations undergo repeated pinning, depinning, and renewed glide as they successively encounter nanoscale precipitates. Other dislocations exhibit persistent local confinement, while dislocation-source activation and dislocation–dislocation interactions occur in the surrounding region.

The dynamic pinning behavior observed here is closely related to the size and spatial distribution of Al_3_Sc precipitates. Statistical analysis of TEM images reveals that the average diameter of Al_3_Sc precipitates is approximately 27 nm, while the average center-to-center inter-particle spacing is approximately 102 nm. Therefore, the effective obstacle spacing experienced by moving dislocations can be estimated as approximately 75 nm after subtracting the precipitate diameter. Considering the nanoscale size and coherent relationship between Al_3_Sc precipitates and the Al matrix, direct shearing of precipitates requires considerable energy, and dislocation bypass through the Orowan mechanism is expected to dominate during deformation.

To quantitatively evaluate the strengthening contribution from precipitate bypass, the critical Orowan shear-stress increment was estimated using [26]:

$$\Delta \tau_{\mathrm{Orowan}\;}=\frac{0.4\mathrm{Gb}}{\pi \sqrt{1-\nu }\lambda }\ln(\frac{2\bar{r}}{b})$$

where *G* is the shear modulus of Al (26 GPa), *b* is the Burgers vector (0.286 nm), and *ν* is Poisson’s ratio (0.33) [27]. The average precipitate radius determined from the TEM statistics is approximately 13.5 nm. Following the stereological correction commonly adopted for spherical precipitates, the mean precipitate radius intersected by the slip plane is estimated as $\bar{r}\;=\;\sqrt{\frac{2}{3}}r\approx \;11.0$ nm. Using the experimentally determined effective inter-particle obstacle spacing of approximately 75 nm, the corresponding critical Orowan shear-stress increment is calculated to be approximately 67 MPa. After conversion to the macroscopic tensile strengthening contribution using the Taylor factor *M* = 3.06 for FCC Al [26], the Orowan strengthening increment is Δσ_Orowan_ = MΔτ_Orowan_ ≈ 205 MPa.

Accordingly, the resistance imposed by Al_3_Sc precipitates is jointly governed by precipitate size, spatial spacing, and the operative dislocation–precipitate interaction mechanism [28,29,30]. Rather than acting merely as immobile barriers that permanently lock dislocations, Al_3_Sc precipitates dynamically strengthen the alloy by increasing the critical stress required for dislocation migration and reducing the effective dislocation mean free path. Similar precipitate shearing, bypassing, and subsequent re-glide behaviors have also been reported in other precipitation-strengthened Al alloys [31]. In addition, dislocations can serve as favorable sites for heterogeneous Al_3_Sc precipitation, further enhancing the structural coupling between precipitates and the retained dislocation network [32]. Therefore, during the early stage of deformation, nanoscale precipitates primarily increase the resistance to intragranular plastic flow by shortening the effective dislocation mean free path, promoting local dislocation storage, and facilitating the activation of dislocation sources.

### 4.2. Precipitate Arrangement and Grain Boundaries Jointly Regulate Dislocation Transport

As deformation approaches a grain boundary, the role of the nanoscale precipitates evolves from local obstacles to spatial regulators of dislocation transport. The nonuniform spatial distribution of Al_3_Sc precipitates produces regions with different effective resistances to slip [33,34]. Consequently, dislocations preferentially migrate through relatively low-resistance paths between neighboring particles, whereas lateral motion toward precipitate-rich regions is restricted. This spatially selective resistance organizes initially dispersed intragranular glide into directional dislocation transport and concentrates dislocation flux toward specific segments of the grain boundary [35].

The response of dislocations after reaching the boundary is governed by the combined effects of the incoming dislocation configuration and the crystallographic mismatch between neighboring grains. Diffraction analysis of adjacent grains confirms a clear crystallographic orientation difference, indicating that the grain boundary interrupts the geometric continuity of the active slip systems. Consequently, a dislocation approaching the boundary may be retained or become entangled when a compatible outgoing slip system is unavailable, whereas transmission becomes possible when the local crystallographic geometry and resolved shear stress are sufficiently favorable [36]. In this respect, the boundary acts simultaneously as a barrier, a dislocation-storage site, and, under appropriate conditions, a transmission interface.

The precipitate distribution and grain boundary thus operate cooperatively rather than independently. The precipitates first reduce the available dislocation mean free path and channel dislocations toward selected boundary regions; the boundary subsequently accommodates the incoming dislocation flux through retention, tangling, absorption, or transmission. Localized delivery of multiple dislocations to a limited boundary segment can increase the local stress concentration, since the stress acting on the leading dislocation in a pile-up increases with the number of accumulated dislocations. This provides a physical basis for the pronounced plastic heterogeneity observed near grain boundaries. Therefore, the intermediate deformation stage represents a transition from precipitate-controlled intragranular strengthening to coupled precipitate–grain-boundary regulation of dislocation transport and strain accommodation.

### 4.3. Precipitate-Mediated Redistribution of Crack-Tip Plasticity and Crack-Path Selection

Near the crack tip, precipitate–dislocation interactions acquire a different physical significance because dislocation motion occurs within a strongly heterogeneous stress field. In ductile Al, crack-tip dislocation emission and glide provide an important mechanism for relaxing the local stress concentration and accommodating crack opening. The presence of nanoscale Al_3_Sc precipitates introduces spatial variations in the resistance to this dislocation motion. Mobile dislocations that overcome local obstacles can transport plastic strain away from the crack tip, whereas strongly pinned dislocations remain confined within restricted regions. The coexistence of mobile and immobilized dislocations therefore produces a heterogeneous plastic zone rather than a uniformly expanding deformation field [25,37].

This selective dislocation mobility provides a physical explanation for crack-tip blunting. Continued dislocation emission and glide locally relax the crack-tip stress concentration and distribute plastic deformation over a broader region, thereby increasing the local curvature of the crack tip. At the same time, precipitate pinning restricts plastic flow along particular directions and forces subsequent dislocation activity to redistribute toward alternative paths [38]. Consequently, precipitates do not simply suppress plastic deformation; instead, they modify where plastic accommodation can occur ahead of the crack.

The same mechanism also provides a basis for crack-path deflection. When repeated dislocation pinning restricts further plastic accommodation along the initially active deformation path, continued loading raises the local driving force for deformation in neighboring regions. Activation of an alternative slip direction then transfers plastic strain away from the original path. Once the newly activated region accommodates deformation more effectively than the precipitate-constrained original region, crack advance preferentially follows this redistributed plastic zone, producing a change in the propagation direction [37,38]. Crack-path deflection should therefore be interpreted as the consequence of competition between locally restricted and newly activated plastic pathways rather than as a direct mechanical deflection of the crack by an individual precipitate.

## 5. Conclusions

The dynamic evolution of precipitate–dislocation interactions in an Al–0.3 wt.% Sc alloy was systematically investigated by in-situ TEM tensile testing with auxiliary EBSD, conventional TEM, EDS, and atomic-resolution HAADF-STEM imaging. The principal conclusions are summarized as follows:

(1) The initial microstructure retains elongated deformation grains, pronounced local orientation gradients, and a high density of dislocations after short-term annealing. The KAM values are predominantly below approximately 2°, with localized peaks reaching approximately 3.5°. Ordered Al_3_Sc precipitates are dispersed throughout the Al matrix, with an average equivalent diameter of 27.0 ± 6.3 nm, an areal number density of ~27.5 μm^−2^, a principal size range of approximately 20–35 nm, and an average center-to-center spacing of approximately 102 nm.
(2) Within grain interiors, the Al_3_Sc precipitates repeatedly interrupt dislocation glide through bowing, pinning, depinning, and renewed motion. After accounting for the precipitate size, the effective obstacle spacing is approximately 75 nm. Using the modified Orowan model, the corresponding critical shear-stress increment is approximately 67 MPa, which corresponds to a macroscopic strengthening contribution of approximately 205 MPa after application of the Taylor factor. These results demonstrate that the strengthening effect arises from dynamic restriction of dislocation transport and reduction in the effective dislocation mean free path rather than permanent immobilization of individual dislocations.
(3) Near grain boundaries, the spatial arrangement of precipitates produces preferential dislocation-transport paths and concentrates dislocation flux toward selected boundary regions. The crystallographic misorientation between adjacent grains further disrupts slip continuity, resulting in distinct boundary responses, including dislocation retention, tangling, absorption, and, where directly observed, transmission into the neighboring grain. The coupled action of precipitates and grain boundaries therefore promotes strongly heterogeneous strain accommodation.
(4) Near the crack tip, selective precipitate pinning redistributes dislocation mobility and local plastic flow. Continued dislocation emission and glide broaden the active plastic region and promote crack-tip blunting, whereas persistent restriction of the initially active pathway favors activation of an alternative slip direction and subsequent crack-path deflection.

Overall, the role of Al_3_Sc precipitates evolves continuously with the local microstructural environment: from quantitative precipitation strengthening within grain interiors, through precipitate–grain-boundary coupling and heterogeneous dislocation transport, to redistribution of crack-tip plasticity and crack-path selection. The results establish a mechanistic connection between nanoscale precipitate characteristics, dislocation transport, local strain heterogeneity, and damage evolution in the Al-Sc alloy.

## Data Availability

The original contributions presented in this study are included in the article. Further inquiries can be directed to the corresponding author.
